# Impact of *Curcuma longa* on Hematopoiesis and Splenic Mass in an Animal Model Undergoing Docetaxel Chemotherapy

**DOI:** 10.3390/biology15030246

**Published:** 2026-01-28

**Authors:** Isabella Morais Tavares Huber, Emerson Luiz Botelho Lourenço, Salviano Tramontin Bellettini, Guilherme Donadel, João Francisco Velasquez Matumoto, Sandra Marisa Pelloso, Maria Dalva de Barros Carvalho, Stéfane Lele Rossoni, Mariana Morais Tavares Colferai, Diego Ricardo Colferai, Roberto Kenji Nakamura Cuman, Leonardo Garcia Velasquez

**Affiliations:** 1 Department of Graduate Studies Coordination Office (COPG), Universidade Paranaense (UNIPAR), Umuarama 87502-210, Paraná, Brazil; 2 PhD Program in Animal Science with Emphasis on Bioactive Products, Universidade Paranaense (UNIPAR), Umuarama 87502-210, Paraná, Brazil; emerson@prof.unipar.br (E.L.B.L.); salviano@prof.unipar.br (S.T.B.); donadel425@gmail.com (G.D.); 3 Department of Oncology, Hematology and Transfusion, Kantonsspital Aarau, Tellstrasse 25, 5001 Aarau, Switzerland; 4 Candido Garcia Foundation, Umuarama 87502-210, Paraná, Brazil; 5 Undergraduate Program in Pharmacy, Universidade Paranaense (UNIPAR), Umuarama 87502-210, Paraná, Brazil; jmatumotovelasquez@gmail.com; 6 PhD Program in Health Sciences, Universidade Estadual de Maringá (UEM), Maringá 87020-900, Paraná, Brazil; smpelloso@gmail.com (S.M.P.); mdbcarvalho@gmail.com (M.D.d.B.C.); ste.tistics@gmail.com (S.L.R.); rkncuman@uem.br (R.K.N.C.); 7 Department of Dermatology, Municipal Health Authority of Apucarana, Apucarana 86800-260, Paraná, Brazil; mari.mtavares@gmail.com (M.M.T.C.); diegocolferai@gmail.com (D.R.C.); 8 PhD Program in Phytotherapy and Medicinal Plants in Primary Health Care, Universidade Paranaense (UNIPAR), Umuarama 87502-210, Paraná, Brazil; leo@prof.unipar.br

**Keywords:** *Curcuma longa*, chemotherapy, hematological parameters, cytoprotection, spleen-associated hematopoiesis

## Abstract

(1) Background: Chemotherapy-induced hematological toxicity remains a major limitation to treatment continuity. Docetaxel is widely used in solid tumors due to its clinical efficacy, despite cumulative bone marrow suppression and splenic alterations. *Curcuma longa* is a phytochemical with antioxidant and anti-inflammatory properties that may confer cytoprotective effects on hematopoietic tissues. (2) Methods: One hundred and five male Wistar rats were randomly allocated into five treatment groups and evaluated at 7, 14, and 21 days. Animals received placebo, docetaxel alone, or docetaxel combined with *Curcuma longa* at doses of 25, 50, or 500 mg/kg/day. Post-treatment hematological parameters and relative spleen weight were analyzed using one-way ANOVA and Tukey’s post hoc test. (3) Results: Docetaxel induced progressive reductions in red blood cell count, hemoglobin, hematocrit, leukocytes, and platelets, with greater severity at day 21. *Curcuma longa* co-treatment partially mitigated these alterations in a dose- and time-dependent manner. Intermediate doses (25–50 mg/kg) showed the most consistent hematoprotective effects. High-dose treatment (500 mg/kg) was associated with no statistically significant change in relative spleen weight. (4) Conclusions: *Curcuma longa* partially mitigated docetaxel-induced hematological toxicity and modulated splenic responses in this experimental model. These findings support further translational studies on chemotherapy-induced hematological toxicity to clarify the role of *Curcuma longa* as a low-toxicity strategy.

## Simple Summary

Chemotherapy with docetaxel is widely used in cancer treatment, but it is frequently associated with significant systemic toxicity, which can compromise patient tolerance and treatment outcomes. Natural compounds with anti-inflammatory and antioxidant properties have been investigated as potential adjuvants to reduce these adverse effects. *Curcuma longa*, a medicinal plant rich in curcuminoids, has demonstrated protective effects in several experimental models of tissue injury and oxidative stress. In this study, we evaluated the protective effects of *Curcuma longa* on hematological parameters and organ toxicity in an experimental animal model treated with docetaxel. Wistar rats were exposed to docetaxel, with or without *Curcuma longa* supplementation, and were evaluated at different time points. Our results show that *Curcuma longa* attenuated docetaxel-induced alterations in blood parameters and reduced systemic toxicity without compromising safety. These findings suggest that *Curcuma longa* may represent a promising adjuvant strategy to mitigate chemotherapy-induced toxicity, supporting further investigation of its potential role in oncology supportive care.

## 1. Introduction

Cancer remains a leading global health burden, with over 20 million new cases and 9.7 million cancer-related deaths reported in 2022, according to the World Health Organization (WHO) [1]. Despite advances in precision oncology, cytotoxic chemotherapy remains a mainstay in the treatment of solid tumors. However, chemotherapy-induced adverse effects, particularly hematological toxicity, significantly impact patient outcomes, often leading to dose reductions, treatment delays, and increased morbidity [2]. Importantly, hematological toxicity represents not only a frequent adverse event but also a critical biological bottleneck that compromises dose intensity, long-term treatment efficacy, and ultimately patient survival, underscoring its clinical relevance and urgency [3,4].

Docetaxel, a widely used taxane-based chemotherapeutic agent, is associated with high rates of myelosuppression and splenic alterations, which compromise hematopoietic function [3,4]. Mechanistically, docetaxel stabilizes microtubules and disrupts mitotic spindle dynamics, preferentially affecting rapidly proliferating cells such as hematopoietic progenitors in the bone marrow [3,4]. Neutropenia, anemia, and thrombocytopenia are among the most common complications, frequently resulting from direct toxicity to bone marrow and hematopoietic organs [3,4,5]. Under conditions of sustained hematopoietic stress, secondary alterations in the spleen may occur, reflecting compensatory immune activation or extramedullary hematopoiesis [3,4,5]. These effects are particularly concerning given the cumulative nature of docetaxel toxicity and its narrow therapeutic index [4,5].

In recent years, natural compounds with anti-inflammatory and antioxidant properties have gained attention for their potential to mitigate chemotherapy-induced toxicity. *Curcuma longa* L. (turmeric) and its principal bioactive constituent, curcumin, have demonstrated cytoprotective effects in various experimental models [6,7,8,9]. Structurally, curcumin belongs to the class of linear diarylheptanoids, a broader group of plant secondary metabolites characterized by two aromatic rings connected by a seven-carbon linker. As explicitly detailed by Sudarshan et al., diarylheptanoids encompass multiple structural subclasses and display considerable chemical diversity, which has been associated with a wide range of reported biological activities, including anti-inflammatory, antioxidant, and immunomodulatory effects across different plant families, notably within the *Zingiberaceae*, to which *C. longa* belongs [10].

While a substantial portion of the literature focuses on isolated curcumin, whole *Curcuma longa* preparations contain multiple bioactive compounds and structurally related diarylheptanoids that may exert synergistic or modulatory effects, which is relevant for translational interpretation and justifies their investigation as therapeutic interventions [8,9,10,11,12,13]. Preclinical studies have shown that curcumin may protect against chemotherapy-related organ damage by modulating oxidative stress, inflammation, and apoptosis [8,9,10,11,12,13]. Furthermore, combination strategies involving phytochemicals and cytotoxic agents have shown promise in enhancing antitumor efficacy while minimizing systemic toxicity [14,15,16,17]. Despite these limitations, important limitations related to curcumin and *Curcuma longa*, including bioavailability, pharmacokinetics, and dose translation from animal models to humans, remain subjects of ongoing debate and warrant cautious interpretation.

**Figure 1:**
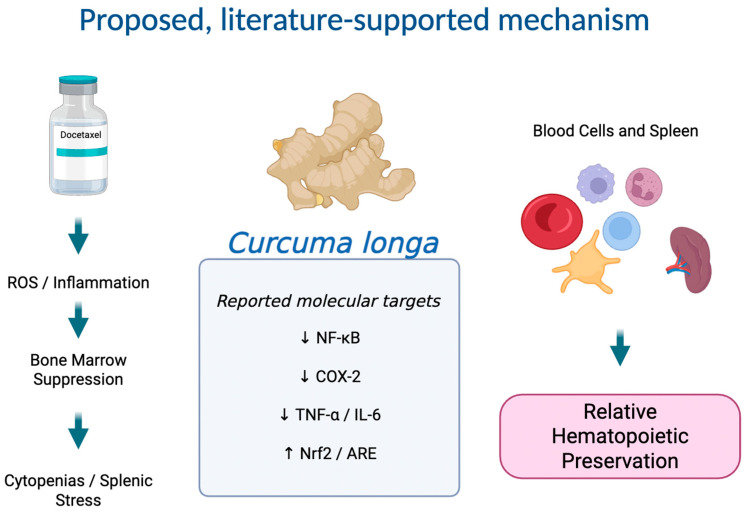
Proposed schematic overview of docetaxel-induced hematological toxicity and the putative modulatory effects of *Curcuma longa*. Docetaxel induces oxidative stress and inflammatory signaling pathways that contribute to bone marrow suppression, cytopenias, and splenic stress. Bioactive compounds from *Curcuma longa*, particularly curcuminoids, have been reported to modulate antioxidant (Nrf2/ARE) and inflammatory (NF-κB, COX-2, cytokine signaling) pathways, potentially contributing to hematopoietic preservation. This schematic represents a literature-supported, hypothesis-generating biological framework.

In this context, the potential of *Curcuma longa* to attenuate docetaxel-induced hematological toxicity and splenic morphological alterations remains poorly understood in in vivo models. Given the spleen’s central role in immune regulation and its capacity to support extramedullary hematopoiesis under conditions of bone marrow suppression, its evaluation alongside hematological parameters is biologically justified in the context of chemotherapeutic stress. Therefore, the present study aimed to investigate the effects of *Curcuma longa* on hematopoiesis and splenic structure in a Wistar rat model undergoing docetaxel chemotherapy. We hypothesized that *Curcuma longa* administration would attenuate docetaxel-induced hematological toxicity and modulate splenic responses in a dose- and time-dependent manner (Figure 1). This research seeks to explore whether phytotherapeutic intervention can preserve hematopoietic function and reduce structural damage in key immune organs during cytotoxic treatment.

## 2. Materials and Methods

This controlled experimental animal study involved 105 male Wistar rats (*n* = 7 per subgroup; 8 weeks of age, healthy animals weighed 250–300 g at baseline), obtained from the Animal Facility of Colombo, Paraná, Brazil. All eligible subjects meeting baseline age and weight criteria were included in the study and were randomly allocated into three primary treatment groups based on duration: Group A (7 days), Group B (14 days), and Group C (21 days). These time points were selected to capture early, intermediate, and cumulative hematological effects of docetaxel, considering its pharmacodynamics and the kinetics of bone marrow suppression and recovery described in previous preclinical studies. Each primary group was subdivided into five treatment, as summarized in Table 1, and described briefly below:

The number of animals per subgroup (*n* = 7) was defined based on previous experimental studies employing similar designs, which demonstrated that this sample size is sufficient to detect hematological and organ-related alterations. Although no formal a priori power calculation was performed, this sample size was considered adequate for an exploratory preclinical study, assuming moderate effect sizes consistent with prior literature. The experimental unit was the individual animal (one rat).

Animals were randomly assigned to groups using a computer-generated randomization sequence. Cage location and handling order were kept constant to minimize potential confounding factors. No animals were excluded, and no inclusion or exclusion criteria beyond baseline health, age, and weight were applied. No specific environmental enrichment was provided beyond standard housing conditions, his approach was adopted to minimize environmental variability that could interfere with hematological outcomes.

Blinding was not feasible during treatment administration; however, outcome assessors and data analysts were blinded to group allocation. The study complied with international ethical guidelines for animal experimentation, adhering to the principles of the 3Rs (Replacement, Reduction, and Refinement).

The docetaxel dose for rats was extrapolated from a human reference dose using allometric scaling based on specific metabolic rate (SMR), according to the methodology proposed by Pachaly [18]. This approach accounts for interspecies differences in metabolic demand and is commonly applied to improve biological relevance in preclinical toxicology models.

A single-dose docetaxel model was selected to isolate acute and subacute hematological toxicity while avoiding cumulative systemic effects that could confound the evaluation of *Curcuma longa*–mediated protection.

Based on this framework, SMR values were calculated for humans (70 kg) and rats (300 g) as follows: SMR_human = 24 and SMR_rat = 95. The human equivalent dose (HED) was set at 0.63 mg/kg and adjusted according to the SMR ratio, resulting in a calculated rat dose of 2.5 mg/kg (0.63/24 × 95).

Accordingly, docetaxel was administered intraperitoneally at a final dose of 2.5 mg/kg, in a volume not exceeding 1 mL/kg. For example, a 250 g rat received 0.625 mg of docetaxel. Importantly, a dose of 2.5 mg/kg docetaxel was selected to induce measurable hematological toxicity while preserving animal survival and experimental interpretability, consistent with commonly applied preclinical chemotherapy toxicity models. This dosing strategy is therefore considered appropriate for the assessment of chemotherapy-induced hematological toxicity in the present study.

The *Curcuma longa* extract (95% curcuminoids) used in this study consisted of a bioavailable formulation (CUREIT^®^) obtained from Farmácia Farmavida (Umuarama, Paraná, Brazil). According to the supplier-provided certificate of analysis, the extract was derived from *Curcuma longa* rhizomes sourced from India, with internal batch number 24D11-B013-207973 and manufacturer batch 2402071001. The material was manufactured on 7 February 2024, with an established expiration date of 6 February 2026, and underwent analytical quality control on 17 May 2024. Throughout the experimental period, the extract was stored under controlled conditions at temperatures up to 25 °C, as specified in the certificate of analysis, ensuring stability and batch traceability. The extract was administered by gavage in doses of 25, 50, or 500 mg/kg/day, diluted in 1 mL/kg of water. The inclusion of a high-dose group (500 mg/kg/day) was intended to explore potential dose-dependent effects and safety boundaries reported in prior toxicological studies.

The chemotherapeutic agent used in this study was docetaxel (Doceglennu^®^, Glenmark Pharmaceuticals, Mumbai, India, 80 mg/4 mL; lot 31170041). The compound was prepared immediately before administration and delivered via intraperitoneal injection at a dose of 2.5 mg/kg, calculated as described above using allometric scaling. The final administration volume did not exceed 1 mL/kg to ensure safety and consistency across all treated subgroups.

Animals were housed in polypropylene cages in a temperature-controlled room (22 ± 2 °C) with a 12-h light/dark cycle and had ad libitum access to standard chow and water throughout the experimental period. After treatment administration, they were placed on a flat surface and observed continuously for 1 h, then hourly for the first 6 h, and subsequently once daily or 7, 14, or 21 days, according to the respective group, with the aim of analyzing their behavior. Behavioral monitoring included locomotion, grooming behavior, feeding activity, and signs of distress during scheduled observation periods. At the end of each experimental period, animals were fasted for 12 h (with access to water), anesthetized using isoflurane, and euthanized via decapitation in accordance with the euthanasia guidelines of the Brazilian CONCEA (2015) [19].

Blood samples for complete blood count (CBC) analysis were collected via retro-orbital plexus puncture under isoflurane anesthesia, a technique selected to ensure sufficient sample volume, with measures taken to minimize pain and tissue damage. The spleen was harvested and weighed for the calculation of relative organ weight, which was determined by the ratio of organ weight (g) to final body weight (g) of the respective animal and expressed as a percentage, according to the following formula: Relative Weight (%) = (Organ Weight/Final Body Weight) × 100. This ratio was selected as a quantitative surrogate marker of splenic response to hematopoietic stress and potential extramedullary hematopoiesis, expressed as a percentage of final body weight.

The primary outcome measure was the complete blood count (CBC), particularly parameters related to hematopoiesis. Secondary outcomes included relative spleen weight. Procedures were selected to model docetaxel-induced hematotoxicity and to evaluate the potential protective effects of *Curcuma longa*. No unexpected adverse events were observed during the experimental period. Humane endpoints were not required, as no animals exhibited signs of severe distress requiring early euthanasia.

Carcasses were stored at −20 °C in labeled plastic bags until collected by a certified biological waste disposal company. Samples were homogenized and stored at 4 °C until analysis.

All procedures were approved by the Institutional Animal Care and Use Committee (IACUC) of University Paranaense under protocol number 40130, and conducted in compliance with the guidelines of the Brazilian CONCEA (2015) [19]. No protocol registration was performed prior to the commencement of this study.

Statistical analysis was performed using one-way analysis of variance (ANOVA) conducted separately for each experimental time point (7, 14, and 21 days), followed by Tukey’s post hoc test for multiple comparisons. A *p*-value < 0.05 was considered statistically significant. Normality and variance homogeneity were tested using Shapiro–Wilk and Levene tests. All analyses were conducted using R Core Team (2025) version 4.5.0. Although a two-way ANOVA could formally assess treatment × time interactions, separate one-way ANOVA analyses were applied at each time point due to the exploratory nature of the study, the limited sample size per subgroup, and the primary objective of identifying time-specific hematological patterns. This analytical choice was therefore considered appropriate for the aims of the present study.

During manuscript preparation, ChatGPT (OpenAI, GPT-5.2) was used exclusively for language refinement, grammar correction, and improvement of scientific clarity. No AI tools were used for study design, data collection, data analysis, data interpretation, or figure generation.

## 3. Results

A time-dependent evaluation of hematological parameters was performed with the primary objective of comparing (I) docetaxel-treated animals versus controls and (II) docetaxel-only groups versus animals receiving *Curcuma longa* at doses of 25, 50, and 500 mg/kg across the 7-, 14-, and 21-day time points. This analytical framework allowed the identification of both time-driven trends and treatment-related effects associated with *Curcuma longa* supplementation.

### 3.1. General Analysis

The ANOVA test, conducted at a 5% significance level, revealed differences among groups for Erythrocytes, Hemoglobin, Mean Corpuscular Hemoglobin Concentration (MCH), Red Cell Distribution Width (RDW), Leukocytes, and Neutrophils. These pooled analyses primarily reflect temporal changes associated with cumulative docetaxel exposure rather than treatment-specific effects, whereas no significant differences were observed for Hematocrit, Mean Corpuscular Volume (MCV), Mean Corpuscular Hemoglobin Concentration (MCHC), Lymphocytes, Monocytes, Platelets, Mean Platelet Volume (MPV), or Spleen body weight.

Tukey’s post hoc test demonstrated that Erythrocyte and Hemoglobin levels were higher at 14 days but declined at 21 days. This biphasic pattern may reflect a transient compensatory hematopoietic response followed by cumulative myelotoxicity associated with prolonged docetaxel exposure. MCH values were lower at 14 days compared to 7 days, while RDW showed a progressive increase up to 21 days, consistent with increasing erythrocyte heterogeneity and marrow stress over time.

Regarding Leukocytes, higher values were observed in the 14-day group compared to the 21-day group, while Neutrophil proportions were higher in the 14-day group than in the 7-day group (Table 2). Although platelet counts and spleen body weight did not reach statistical significance in this global analysis, this may reflect high interindividual variability and limited statistical power, rather than the absence of biologically relevant effects.

### 3.2. Analysis by Period

To better characterize treatment-specific effects, group-wise analyses were conducted separately at each time point, comparing control animals (GC), docetaxel-only groups (GQ), and *Curcuma longa*–treated groups at 25 mg/kg (G25), 50 mg/kg (G50), and 500 mg/kg (G500).

At 7 days, statistically significant differences were observed only for MCHC, with the AG50 group exhibiting lower values compared to the other groups. This isolated finding likely represents an early, transient dose-related effect or biological variability rather than a sustained hematological alteration. No other hematological or splenic parameters differed significantly at this time point.

At 14 days, significant differences were observed for MCH, RDW, Leukocytes, and Neutrophils. Tukey’s test indicated that the BG25 group exhibited lower MCH and RDW values, whereas the BG50 group showed higher Leukocytes and Neutrophils counts compared to the other groups. These findings suggest that intermediate doses of *Curcuma longa* (25–50 mg/kg) may represent a hematoprotective window, particularly with respect to leukopoiesis and innate immune preservation during the intermediate phase of docetaxel exposure.

At 21 days, significant differences were found in MCV, MCH, MCHC, and RDW. Specifically, MCV was higher in the G25 and G50 groups, while the G500, GC, and GQ groups exhibited reduced values. Conversely, MCHC was higher in the G500, GC, and GQ groups compared to G25 and G50. RDW was lower in G500 compared to G25 and G50, whereas MCH was lower in the docetaxel-only group (GQ) relative to G25 (Table 3). These divergent erythrocyte index patterns at 21 days highlight dose-dependent modulation of red cell morphology, with partial attenuation of docetaxel-induced dyserythropoiesis at intermediate *Curcuma longa* doses, while high-dose exposure was associated with greater variability.

Regarding splenic outcomes, relative spleen mass did not differ significantly between groups at any time point; however, marked variability was observed at 21 days, particularly in the G500 group, as reflected by the large standard deviation. This variability suggests heterogeneous splenic responses and may mask subgroup-specific effects in global analyses. No animals were excluded, and no post hoc outlier removal was performed.

Across all experimental periods, leukocyte and platelet parameters were comprehensively assessed. The absence of consistent statistically significant differences should be interpreted cautiously, given the known sensitivity of these parameters to taxane-induced myelotoxicity and the inherent biological variability of hematological responses in preclinical models.

## 4. Discussion

### 4.1. Hematological Toxicity: A Limiting Factor in Oncology

Despite advances in precision oncology, including targeted therapies and immunotherapies, cytotoxic chemotherapy remains the cornerstone of treatment for most solid tumors. However, chemotherapy-associated adverse events (CAAEs) continue to pose a substantial clinical and economic burden, contributing to significant morbidity and mortality, and negatively impacting patients’ physical, emotional, and social well-being. Hematological toxicities such as neutropenia, anemia, and thrombocytopenia are among the most frequent complications, often leading to treatment delays, dose reductions, or discontinuation—factors that may compromise therapeutic efficacy and increase the risk of disease recurrence [1,2,3].

**Figure 2:**
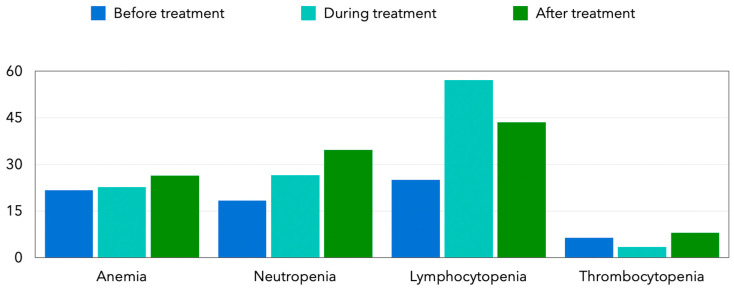
Frequency of hematological abnormalities reported in breast cancer patients before, during, and after chemotherapy. The figure was created by the authors based on data reported in the literature. Data from Aynalem M et al. [3].

Hematological abnormalities are frequently observed in cancer patients, both prior to and following therapeutic interventions (Figure 2) [3]. Pre-treatment hematological alterations are largely attributed to bone marrow suppression secondary to neoplastic infiltration, which compromises hematopoietic function [3,4]. Numerous studies have reported a high incidence of baseline anemia, neutropenia, and thrombocytopenia in oncology populations. Post-treatment hematological derangements, in contrast, may arise not only from the continued impact of the malignancy but also from the cytotoxicity associated with chemotherapy and radiotherapy regimens [3,4,5].

Among these abnormalities, anemia is the most prevalent and is associated with multiple underlying mechanisms, including chronic tumor-related bleeding, direct bone marrow infiltration, tumor-induced malnutrition, altered iron metabolism, renal impairment, and general suppression of hematopoiesis. Leukopenia—especially neutropenia—is primarily linked to the myelosuppressive nature of antineoplastic agents [3,4,5]. Thrombocytopenia, on the other hand, is often attributed to tumor-mediated activation of the coagulation cascade, resulting in increased platelet consumption. Conversely, thrombocytosis has been identified as a negative prognostic marker, correlating with reduced progression-free survival and worse overall outcomes in various malignancies [3,7].

In light of these challenges, there is a growing demand for integrative therapeutic strategies capable of attenuating chemotherapy-induced toxicity without diminishing its antitumor potential. Within this context, phytochemicals such as *Curcuma longa* have emerged as promising adjuvants, owing to their anti-inflammatory, antioxidant, and cytoprotective properties demonstrated in preclinical models [8].

### 4.2. The Therapeutic Promise of Curcuma longa

Growing preclinical and clinical evidence has demonstrated that curcumin and its structural analogs possess a broad spectrum of pharmacological properties, including potent antioxidant, anti-inflammatory, and antineoplastic activities. Among these, its anticancer potential is particularly noteworthy. Curcumin has been shown to induce apoptosis and exert antiproliferative effects across a wide range of tumor cell lines, such as those derived from prostate, breast, colorectal, pancreatic, and renal cancers. Notably, doses up to 12 g/day administered over 3 months have been reported as safe in humans [8,9].

Mechanistically, curcumin modulates several oncogenic and tumor-suppressor pathways. It inhibits telomerase reverse transcriptase activity and downregulates anti-apoptotic proteins such as Bcl-2 [8,9,11]. At the molecular level, curcumin interacts with key regulators of angiogenesis, metastasis, and cell survival, and interferes with dysregulated signaling cascades including PI3K/Akt and NF-κB. It also modulates crucial cellular processes through its effects on p53, MAPKs, PTEN, and microRNAs. NF-κB, a transcription factor implicated in both inflammation and tumorigenesis, is a major molecular target of curcumin. Under pathological stimuli—such as cytokines, carcinogens, free radicals, or ionizing radiation—NF-κB is upregulated via TNF-α-mediated activation of the IκB kinase (IKK) complex. Curcumin effectively inhibits this process by suppressing IKK activity, thereby preventing NF-κB nuclear translocation and downstream gene expression [8,12,13].

In both in vitro and in vivo models, curcumin has demonstrated cytotoxicity against pancreatic cancer cells through the suppression of oxidative stress and angiogenesis, and through the induction of programmed cell death. Collectively, these data reinforce curcumin’s role as a promising adjuvant compound in oncology, capable of targeting multiple hallmarks of cancer with a favorable safety profile [8,13].

Recent preclinical data suggest that combination regimens integrating natural compounds and cytotoxic agents may enhance antineoplastic efficacy without exacerbating systemic toxicity [14]. Docetaxel, a microtubule-stabilizing agent approved at doses of 30 to 75 mg for the treatment of metastatic castration-resistant prostate cancer (mCRPC), remains a cornerstone of standard therapy [15]. Nonetheless, prolonged exposure to docetaxel has been associated with significant cumulative toxicity, often limiting treatment adherence and compromising patient outcomes [16].

### 4.3. Synergism Between Curcumin and Docetaxel

In this context, Banerjee et al. [17] demonstrated that the co-administration of curcumin (20 µM) and docetaxel (10 nM) significantly enhanced cytotoxicity in prostate cancer cell lines (DU145 and PC-3), compared to monotherapy with either agent. The combined treatment potentiated apoptosis and markedly suppressed cell proliferation through the downregulation of key oncogenic pathways, including COX-2, phospho-Akt, PI3K, NF-κB, p53, and receptor tyrosine kinases (RTKs). These findings highlight the mechanistic synergy between curcumin and docetaxel, suggesting that phytochemical-based adjuvants may augment therapeutic efficacy while potentially attenuating chemotherapy-induced toxicity and overcoming resistance mechanisms in prostate cancer.

Given the substantial burden of hematological toxicity associated with cytotoxic chemotherapy, particularly with agents such as docetaxel, this study aimed to evaluate the extent of hematological alterations induced by docetaxel and the potential mitigating role of *Curcuma longa* as an adjuvant therapeutic agent. The proposed hypothesis is that *Curcuma longa*, owing to its known anti-inflammatory and antioxidant properties, may contribute to the preservation of hematopoietic integrity and attenuation of myelosuppressive effects when used in combination with standard chemotherapy. This investigation seeks to contribute to the growing body of evidence supporting the integration of phytotherapeutic agents into oncologic care as a complementary strategy to improve treatment tolerability and protect physiological homeostasis.

### 4.4. Hematological Preservation in Docetaxel-Treated Animals

In light of these challenges, the present study investigated the potential adjuvant role of *Curcuma longa* in modulating hematologic toxicity induced by docetaxel. The results demonstrated a time- and dose-dependent trend toward hematological preservation associated with *Curcuma longa* co-treatment, affecting key parameters such as red blood cell count, hemoglobin concentration, hematocrit, white blood cell count, and platelet levels. These findings are in agreement with preclinical evidence indicating that curcumin—*Curcuma longa*’s principal bioactive component—possesses anti-inflammatory and antioxidant properties capable of mitigating chemotherapy-induced damage [9,17,18,19].

Notably, a tendency toward attenuation of leukopenia and thrombocytopenia, particularly at intermediate doses (25–50 mg/kg), may indicate a protective effect on myeloid and megakaryocytic lineages. This effect may be partially mediated by antioxidant and anti-inflammatory mechanisms previously described, which are implicated in cytotoxic drug resistance and hematopoietic regulation [12,13,17].

### 4.5. Splenic Response and Immunomodulation

The analysis of relative spleen weight in this study revealed no statistically significant differences among treatment groups across all timepoints. However, a non-significant tendency toward increased spleen mass was observed at day 21, particularly in the group receiving 500 mg/kg of *Curcuma longa*, accompanied by marked interindividual variability. Although these findings did not reach statistical significance, the observed trend may reflect exploratory signals of compensatory responses, such as extramedullary hematopoiesis or altered reticuloendothelial activity in response to docetaxel-induced bone marrow suppression.

Previous studies have reported that high-dose curcumin can stimulate the production of hematopoietic cytokines and enhance splenic macrophage activation, phenomena that could underlie the morphological changes noted in our model. Nevertheless, given the absence of statistically significant differences and the high variability observed, these results should be interpreted with caution. Further investigations incorporating histomorphological evaluation, immunophenotyping, and cytokine profiling are warranted to clarify whether the observed splenic changes represent adaptive hematopoietic responses or potential immunological overstimulation during prolonged exposure.

Overall, splenic findings should be interpreted as secondary and exploratory, given that relative spleen weight was the sole splenic endpoint assessed and was characterized by high variability and lack of statistical significance. While biologically plausible mechanisms such as extramedullary hematopoiesis and immune cell redistribution have been described in prior models [2,8,11,14,20,21,22,23,24,25,26,27], histological, immunophenotypic, and functional analyses are required to validate whether these processes were truly engaged in the present study.

### 4.6. Red Cell Indices and Erythropoietic Efficiency

Red cell indices, including mean corpuscular volume (MCV), mean corpuscular hemoglobin (MCH), and mean corpuscular hemoglobin concentration (MCHC), remained within physiological ranges but displayed modest elevation in MCH in *Curcuma longa*-treated animals over time. These changes may reflect enhanced hemoglobin synthesis or improved erythrocyte morphology, although further ultrastructural analysis would be required to confirm this.

Red Cell Distribution Width (RDW) is a quantitative index of erythrocyte size heterogeneity and is commonly elevated in conditions associated with ineffective erythropoiesis, increased red cell turnover, or oxidative stress. In this study, RDW levels showed a mild, progressive elevation over time in animals exposed to docetaxel alone, particularly at the 21-day mark, suggesting a disruption in erythroid maturation or red cell membrane integrity likely mediated by oxidative injury to hematopoietic progenitors.

Notably, co-treatment with *Curcuma longa*—especially at 25 and 50 mg/kg doses—was associated with stabilization of RDW values across timepoints, with values remaining within a narrow physiological range. This observation may reflect improved erythrocyte morphology preservation and more synchronized erythropoiesis under adjunctive phytotherapy. These findings are consistent with the reported antioxidant and anti-inflammatory actions attributed primarily to curcumin and related curcuminoids present in *Curcuma longa*, which may contribute to a more favorable bone marrow microenvironment and support erythroid progenitor integrity under cytotoxic stress [13,17,27]

From a mechanistic perspective, antioxidant response pathways relevant to erythroid homeostasis may contribute to the RDW stabilization observed in *Curcuma longa*–treated groups. Modulation of redox balance and inflammation-related signaling by curcumin may indirectly influence erythroid coordination. Accordingly, the reduced RDW variability detected at intermediate doses may serve as an indirect marker of preserved erythropoietic efficiency under cytotoxic stress, supporting the hematoprotective potential of *Curcuma longa* [13,27].

These hematologic findings provide additional insight into the temporal dynamics of bone marrow suppression and potential hematologic recovery under adjunctive phytotherapy. Docetaxel, a microtubule-stabilizing chemotherapeutic agent, is well-documented for inducing dose-limiting myelosuppression, particularly neutropenia and thrombocytopenia, through inhibition of mitotic spindle assembly and consequent apoptosis in proliferating hematopoietic progenitor cells19. In this experimental model, a progressive decline in red blood cell (RBC) count, hemoglobin (Hb), and hematocrit (Ht) was observed in animals exposed solely to docetaxel, with the most significant alterations noted on day 21, confirming the cumulative cytotoxic effect of the agent.

The co-treatment with *Curcuma longa*—especially at doses of 25 and 50 mg/kg—attenuated the extent of RBC, Hb, and Ht reduction over time. This suggests a potential erythropoietic protective effect, which may be partially related to the antioxidative action of curcumin [8,12,13,17,27]. Curcumin has also been shown to modulate hepcidin and iron metabolism, factors critical to erythropoiesis under inflammatory and cytotoxic stress [12,27].

### 4.7. Preservation of Myelopoiesis and Immune Competence Under Chemotherapy Stress

Docetaxel-induced leukopenia, particularly neutropenia, represents a major clinical challenge due to the increased risk of infection. In the present study, leukocyte counts declined over time in docetaxel-only groups, as expected. In contrast, co-treatment with *Curcuma longa*, especially at intermediate doses (25–50 mg/kg), was associated with relative preservation of total leukocyte and neutrophil counts at 14 and 21 days. These findings suggest a potential modulatory effect on myelopoiesis during chemotherapy-induced stress, although intergroup variability warrants cautious interpretation [14,17,27]. Such effects may be partially mediated by the known ability of curcumin to modulate inflammatory cytokines and hematopoietic growth factors implicated in myeloid lineage regulation, as reported in previous experimental models [11,14,17,28]

From a translational perspective, these immune trends support future evaluation of inflammatory biomarkers such as the neutrophil-to-lymphocyte ratio (NLR), a recognized prognostic indicator in several solid tumors, such as lung, colorectal and breast cancer. Assessment of NLR may help clarify whether *Curcuma longa* influences systemic inflammatory balance during chemotherapy and inform its potential clinical applicability [20,21,28].

Overall, the relative preservation of white blood cell counts observed in co-treated animals suggests maintenance of immune competence during cytotoxic exposure, a feature that may be particularly valuable in adjuvant settings where infection risk and antitumor immune surveillance are critical [20,21,22,23,27].

### 4.8. Thrombopoiesis Preservation and Platelet Recovery

In this model, platelet counts exhibited a declining trend under docetaxel monotherapy, consistent with the known myelosuppressive profile of taxanes. Although co-treatment with *Curcuma longa*—particularly at 25 and 50 mg/kg—showed a numerical tendency toward higher platelet counts, no statistically significant differences were detected between groups. Accordingly, while these findings suggest a possible modulatory influence on thrombopoiesis, the present data do not provide conclusive evidence of a protective effect on platelet production.

Mechanistically, curcumin has been reported to protect megakaryocytic progenitors from oxidative stress–induced apoptosis and to modulate cytokine networks involved in megakaryopoiesis [9,11,17,28]. Such mechanisms may underlie the observed trends; however, given the absence of statistical significance and the variability of platelet responses, these interpretations should be regarded as exploratory.

Thrombocytopenia is a common and often dose-limiting adverse effect of cytotoxic chemotherapy, which may lead to bleeding events, need for platelet transfusions, treatment delays, and overall compromise in oncologic outcomes. In this context, even modest attenuation of platelet decline may be clinically relevant; nonetheless, confirmation of a meaningful effect of *Curcuma longa* on thrombopoiesis will require further studies incorporating bone marrow histopathology, megakaryocyte-specific markers (e.g., CD41, CD42b), and functional platelet assessments.

Ultimately, maintaining platelet levels within a functional range can reduce the risk of hemorrhagic complications and minimize interruptions in the treatment regimen—an essential goal in the clinical management of cancer patients receiving chemotherapy.

### 4.9. Comparison with Other Cytotoxic Agents

The hematopoietic alterations observed in this docetaxel-based experimental model are in line with findings reported for other commonly used cytotoxic agents, such as cisplatin and cyclophosphamide. Both agents are well-documented for inducing dose-dependent bone marrow suppression, leading to reductions in peripheral blood cell counts, including erythrocytes, leukocytes, and platelets [28,29,30]. Cisplatin, a platinum-based compound, exerts its cytotoxicity through DNA cross-linking and has been associated with significant anemia and leukopenia in preclinical models, particularly through suppression of erythroid progenitors and oxidative damage to bone marrow stromal cells35. Similarly, cyclophosphamide, an alkylating agent, causes profound and sustained suppression of hematopoiesis via DNA alkylation, with marked effects on lymphocyte and megakaryocyte lineages [29,30].

Notably, several studies investigating phytotherapeutic adjuvants—such as *Curcuma longa*, *Panax ginseng*, and *Camellia sinensis*—have reported hematoprotective effects across different chemotherapy models, highlighting their potential to mitigate oxidative stress and inflammation-mediated marrow suppression [30,31,32]. The temporal patterns of cytopenias and recovery observed in the present docetaxel model bear resemblance to cisplatin and cyclophosphamide studies, reinforcing the construct validity of the experimental framework and suggesting that the protective trends induced by *Curcuma longa* may hold relevance across diverse cytotoxic contexts.

These parallels strengthen the translational relevance of the findings and provide a compelling rationale for broader investigation of *Curcuma longa* as an adjunctive agent in chemotherapy protocols involving various myelotoxic regimens.

### 4.10. In Silico Pathway- and Target-Based Mechanistic Support for the Observed Hematological Outcomes

To provide additional biological context for the functional hematological findings observed in this study, an in silico pathway- and target-based analysis was performed based on curated biological databases and high-quality peer-reviewed literature. This integrative approach aimed to contextualize the experimentally observed hematological and splenic outcomes within previously reported molecular networks associated with *Curcuma longa* and its major bioactive constituent, curcumin [33,34,35].

**Figure 3:**
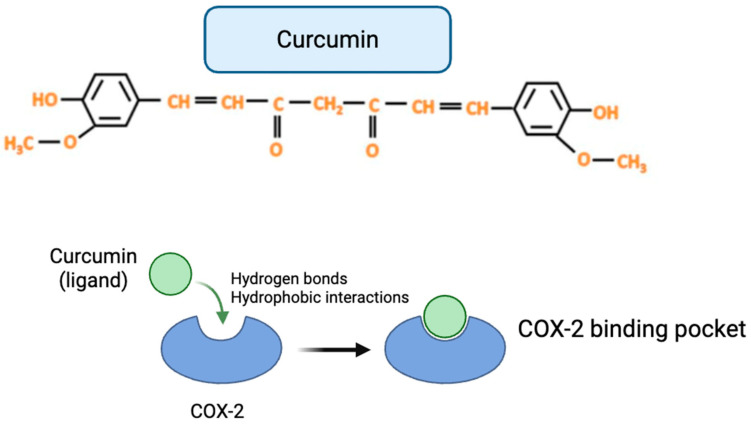
Representative 2D schematic illustration of curcumin interaction with COX-2 based on literature-reported molecular docking studies. The figure depicts a simplified, schematic representation of curcumin (ligand) interacting with the COX-2 binding pocket through generalized hydrogen bonding and hydrophobic interactions, as described in previously published in silico analyses. This original illustration was created for contextual purposes.

As supported by the functional findings of the present study and by previous preclinical evidence, curcumin has been reported to modulate multiple molecular targets involved in oxidative stress regulation, inflammatory signaling, and cellular survival pathways. Among the most consistently reported targets are nuclear factor kappa B (NF-κB), nuclear factor erythroid 2–related factor 2 (Nrf2), cyclooxygenase-2 (COX-2), tumor necrosis factor alpha (TNF-α), interleukin-6 (IL-6), and components of the PI3K/Akt and STAT3 signaling cascades (Figure 3) [33,34,35,36,37,38,39]. These targets are centrally implicated in the biological processes underlying chemotherapy-induced hematological toxicity, including reactive oxygen species (ROS) accumulation, inflammatory cytokine release, and impaired hematopoietic progenitor cell function [32,40,41,42].

From a pathway-level perspective, curated resources such as KEGG, Reactome, STRING, and the Comparative Toxicogenomics Database consistently associate curcumin-related targets with signaling networks involved in antioxidant defense (e.g., Nrf2/ARE pathway), inflammatory response modulation (e.g., NF-κB and cytokine signaling), and stress-responsive hematopoietic regulation [43,44,45,46]. Activation of antioxidant response elements and suppression of pro-inflammatory transcriptional programs have been linked to improved stability of the bone marrow microenvironment and preservation of hematopoietic lineage output in experimental models of cytotoxic injury [32,40,41,42,46]. These mechanisms are biologically consistent with the attenuation of red blood cell heterogeneity (RDW stabilization), partial preservation of leukocyte counts, and maintenance of erythroid indices observed in *Curcuma longa*–treated animals in the present study (Table 4).

Regarding splenic outcomes, although no histopathological or immunophenotypic analyses were performed, prior experimental and in silico evidence suggests that curcumin-associated signaling pathways play roles in immune regulation and adaptive hematopoietic responses under conditions of bone marrow stress [12,46,48]. Molecular targets such as NF-κB and cytokine-mediated signaling nodes are known to participate in splenic immune activation and extramedullary hematopoiesis in response to myelosuppressive stimuli [46,48]. Within this conceptual framework, the variability in relative spleen weight observed at later time points, particularly at higher *Curcuma longa* doses, may be explored in future studies as potential adaptive immune or hematopoietic responses. Nevertheless, definitive confirmation of these interpretations will require future research incorporating dedicated morphological and functional assessments.

Importantly, this in silico pathway- and target-based analysis provides a biologically plausible and integrative framework that contextualizes the functional hematological trends observed experimentally within established molecular pathways known to be modulated by curcumin [32,33,34,35,40,41,42,46]. This integrative perspective supports the interpretation that the hematoprotective patterns identified, particularly at intermediate doses, are consistent with previously reported antioxidant and anti-inflammatory mechanisms, while reinforcing the need for future studies incorporating histopathological, molecular, and cytokine-level analyses to directly validate these pathways.

### 4.11. Translational Implications and Clinical Perspectives

The hematopoietic modulation demonstrated by *Curcuma longa* in this preclinical model carries significant implications for translational oncology. One of the major barriers to optimal chemotherapy delivery in clinical practice is treatment-limiting hematological toxicity, which often necessitates dose reductions, cycle delays, or even early discontinuation. These modifications compromise dose intensity—a key determinant of therapeutic efficacy in many oncologic protocols—and have been associated with inferior outcomes across various solid tumors [2,3,49].

A central and consistent finding of this study is the superior hematological profile observed at intermediate *Curcuma longa* doses (25–50 mg/kg). Across multiple hematological domains, these doses were associated with more stable erythroid and leukocyte parameters compared with both docetaxel-only and high-dose *Curcuma longa* groups, suggesting the existence of an optimal hematoprotective window. In contrast, higher doses were associated with increased variability and potential immune overstimulation, underscoring the importance of dose optimization for translational applications.

The present findings suggest that *Curcuma longa*, through its hematoprotective and immunomodulatory actions, may help preserve bone marrow function during cytotoxic chemotherapy, thereby potentially supporting treatment continuity and adherence to scheduled dosing regimens. This is particularly relevant for agents such as docetaxel, whose clinical benefit is closely linked to adherence to scheduled dosing regimens [15,16,49]. The ability of *Curcuma longa* to attenuate erythrocyte, leukocyte, and platelet suppression—especially during the early and intermediate treatment phases—may reduce the burden of hematological toxicity and warrants further investigation regarding its impact on the need for supportive interventions, such as growth factors or transfusions, in appropriately designed clinical trials.

Furthermore, the observed dose- and time-dependent effects highlight the importance of identifying an optimal therapeutic window that maximizes hematologic support while minimizing the risk of immune overstimulation or organomegaly. These results provide a robust rationale for future clinical trials investigating *Curcuma longa* as an adjuvant agent in cancer patients undergoing chemotherapy. Such studies could assess endpoints including hematologic toxicity profiles, chemotherapy dose intensity, infection rates, and quality of life metrics, offering a holistic view of its clinical utility.

Given its favorable safety profile, low cost, and accessibility, *Curcuma longa* emerges as a promising phytotherapeutic candidate for integration into multimodal oncologic care strategies aimed at improving tolerability and enhancing treatment outcomes.

Overall, these preclinical results support the rationale for future clinical trials investigating *Curcuma longa* as an adjunctive strategy to mitigate hematological toxicity and maintain dose intensity during taxane-based chemotherapy. Although this is a preclinical animal study, the findings may be relevant to human oncology by supporting the investigation of *Curcuma longa* as an adjuvant strategy to mitigate chemotherapy-induced hematological toxicity.

### 4.12. Limitations of the Study

Despite providing valuable insights into the hematoprotective potential of *Curcuma longa*, this study has additional limitations that should be acknowledged. The absence of histopathological analysis of bone marrow and spleen and the lack of cytokine profiling and oxidative stress markers restrict mechanistic interpretation of the observed hematological and splenic changes. In addition, the lack of a *Curcuma longa*–only control group precludes a definitive distinction between intrinsic hematopoietic stimulation and protection against docetaxel-induced toxicity. And finally, the use of a single sex may limit generalizability, given known sex-related differences in hematopoietic and immune responses, and repeated oral gavage and handling represent potential sources of physiological stress, although these procedures were applied consistently across all groups. It is important to mention that the highest dose was included as an exploratory arm and should not be directly extrapolated to humans, particularly given the known limitations of curcumin bioavailability. Future studies addressing these limitations will be essential to validate and extend the present findings.

## 5. Conclusions

This study provides strong preclinical evidence that *Curcuma longa* exerts time- and dose-dependent hematoprotective effects in an experimental model of docetaxel-induced myelosuppression in rats. Adjuvant administration of *Curcuma longa*, particularly at intermediate doses (25–50 mg/kg), was associated with preservation of erythroid, leukocytic, and megakaryocytic parameters, attenuating the cytopenias typically observed with docetaxel monotherapy.

Furthermore, the observed stabilization of red cell distribution width (RDW) and the dose- and time-dependent changes in splenic morphology suggest a possible modulation of hematopoietic dynamics, which may reflect broader regulatory effects on hematopoietic homeostasis. These interpretations remain hypothesis-generating, as direct assessment of bone marrow architecture and functional immune responses was not performed, and the proposed involvement of antioxidant, anti-inflammatory, and immunomodulatory mechanisms should therefore be regarded as mechanistic speculation rather than demonstrated causality.

The pronounced splenomegaly observed at higher doses and longer exposure periods raises relevant considerations regarding safety limits and the risk of immune hyperstimulation. These findings underscore the importance of defining an optimal therapeutic window for phytotherapeutic interventions in the context of chemotherapy.

Collectively, the results of this study support the potential of *Curcuma longa* as a low-toxicity adjuvant capable of enhancing hematological resilience during cytotoxic treatment. The translational implications of this research warrant further investigation, including mechanistic studies, histopathological analyses of the bone marrow and spleen, and carefully designed clinical studies in oncological settings, with the ultimate goal of improving treatment tolerability and preserving dose intensity in cancer therapy.

## Figures and Tables

**Table 1:** Groups of treatment.

Group	Duration (Days)	Treatment	*Curcuma longa* Dose (mg/kg/day)	*n*
A1	7	Placebo	–	7
A2	7	Docetaxel	–	7
A3	7	Docetaxel + *Curcuma longa*	25	7
A4	7	Docetaxel + *Curcuma longa*	50	7
A5	7	Docetaxel + *Curcuma longa*	500	7
B1	14	Placebo	–	7
B2	14	Docetaxel	–	7
B3	14	Docetaxel + *Curcuma longa*	25	7
B4	14	Docetaxel + *Curcuma longa*	50	7
B5	14	Docetaxel + *Curcuma longa*	500	7
C1	21	Placebo	–	7
C2	21	Docetaxel	–	7
C3	21	Docetaxel + *Curcuma longa*	25	7
C4	21	Docetaxel + *Curcuma longa*	50	7
C5	21	Docetaxel + *Curcuma longa*	500	7

**Table 2:** ANOVA e Tukey’s post hoc test for multiple comparison of hematological parameters and spleen body weight.

Parameters	Variable	A—7 Days	B—14 Days	C—21 Days	*p* Value
Hematological	MCHC	22.82 ± 2.33 a	23.43 ± 0.56 a	22.71 ± 1.47 a	0.1408
	MCH	15.71 ± 0.68 a	15.06 ± 0.94 b	15.38 ± 0.78 ab	0.0050
	Hematocrit	56.56 ± 3.6 a	59.11 ± 2.67 a	57.03 ± 10.67 a	0.2401
	Hemoglobin	13.32 ± 0.94 ab	13.71 ± 0.67 a	12.59 ± 2.14 b	0.0045
	Erythrocytes	8.45 ± 0.58 ab	8.82 ± 0.48 a	8.06 ± 1.3 b	0.0020
	Leukocytes	13,068.07 ± 4453.45 ab	15,214.29 ± 5244.39 a	11,570.59 ± 2972.25 b	0.0030
	Lymphocytes	65.74 ± 7.31 a	60.29 ± 11.17 b	63.21 ± 9.03 ab	0.0567
	Monocytes	4.03 ± 1.82 a	4 ± 1.7 a	3.85 ± 1.4 a	0.8939
	Neutrophils	30 ± 6.16 b	35.14 ± 10.86 a	32.74 ± 7.96 ab	0.0492
	Platelets	160,959.12 ± 192,933.5 a	134,289.14 ± 34,126.59 a	123,678.53 ± 35,142.58 a	0.3865
	RDW	14.88 ± 1.34 b	15.63 ± 1 a	15.85 ± 1.23 a	0.0031
	MCV	66.29 ± 3.71 a	66.6 ± 3.11 a	68.29 ± 5.1 a	0.0948
	MPV	6.38 ± 0.42 a	6.59 ± 0.53 a	6.5 ± 0.42 a	0.1593
Body weight	Spleen	1.11 ± 0.21 a	1.02 ± 0.24 a	2.79 ± 10.13 a	0.3673

Note: Values are expressed as mean ± standard deviation of the mean. Mean differences were evaluated using ANOVA followed by Tukey’s post hoc test. Different lowercase letters (a, b) within the same row indicate statistically significant differences between groups (*p* < 0.05), whereas values sharing at least one common letter do not differ significantly.

**Table 3:** ANOVA and Tukey’s post hoc test for multiple comparison of hematological parameters and spleen body weight.

Period	Variable	G25	G50	G500	GC	GQ	*p* Value
7 days							
	Hematological Parameters						
	MCHC	23.43 ± 0.79 a	19.83 ± 4.58 b	23.57 ± 0.53 a	23.14 ± 0.38 a	23.71 ± 0.49 a	0.01
	MCH	15.43 ± 0.98 a	16 ± 0.63 a	15.86 ± 0.38 a	15.57 ± 0.79 a	15.71 ± 0.49 a	0.59
	Hematocrit	55.86 ± 2.12 a	57.83 ± 3.06 a	55.43 ± 6.63 a	56.86 ± 2.85 a	57 ± 1.73 a	0.78
	Hemoglobin	13.29 ± 0.76 a	13.67 ± 0.82 a	13.14 ± 1.68 a	13.14 ± 0.69 a	13.43 ± 0.53 a	0.86
	Erythrocytes	8.43 ± 0.47 a	8.68 ± 0.35 a	8.21 ± 1.04 a	8.42 ± 0.51 a	8.54 ± 0.26 a	0.70
	Leukocytes	12,371.43 ± 1953.39 a	12,552.42 ± 7812.95 a	16,514.29 ± 5108.63 a	11,214.29 ± 2193.5 a	12,614.29 ± 2133.41 a	0.22
	Lymphocytes	64.86 ± 8.36 a	71.67 ± 2.88 a	64.71 ± 7.32 a	63.71 ± 7.11 a	64.57 ± 8.34 a	0.31
	Monocytes	4 ± 1.83 a	3.17 ± 0.75 a	4.29 ± 2.14 a	4.43 ± 2.64 a	4.14 ± 1.35 a	0.78
	Neutrophils	30.86 ± 6.72 a	25.17 ± 2.4 a	30.86 ± 6.2 a	31.57 ± 6.35 a	30.86 ± 7.17 a	0.35
	Platelets	117,628.57 ± 65,491.7 a	263,883.33 ± 331,829.42 a	106,742.86 ± 46,057.97 a	225,244.29 ± 282,297.4 a	106,000 ± 48,048.1 a	0.43
	RDW	14.71 ± 1.6 a	14.67 ± 2.25 a	15.57 ± 1.27 a	14.71 ± 0.49 a	14.71 ± 0.76 a	0.70
	MCV	66.57 ± 3.55 a	63.17 ± 6.55 a	67.57 ± 2.07 a	67.43 ± 2.07 a	66.29 ± 2.29 a	0.21
	MPV	6.11 ± 0.23 a	6.38 ± 0.28 a	6.69 ± 0.54 a	6.53 ± 0.47 a	6.19 ± 0.28 a	0.05
	Body Weight Parameters						
	Spleen	1.16 ± 0.22 a	1.05 ± 0.17 a	1.18 ± 0.23 a	1.11 ± 0.27 a	1.06 ± 0.15 a	0.7046
14 days							
	Hematological Parameters						
	MCHC	23.29 ± 0.49 a	23.71 ± 0.49 a	23.71 ± 0.49 a	23.29 ± 0.76 a	23.14 ± 0.38 a	0.1652
	MCH	13.86 ± 0.69 b	15.71 ± 0.76 a	15.29 ± 0.49 a	15 ± 0.58 a	15.43 ± 0.98 a	<0.001
	Hematocrit	59.29 ± 1.38 a	58 ± 2.16 a	58.71 ± 2.87 a	59.14 ± 3.34 a	60.43 ± 3.26 a	0.5621
	Hemoglobin	13.71 ± 0.49 a	13.43 ± 0.53 a	13.86 ± 0.69 a	13.71 ± 0.76 a	13.86 ± 0.9 a	0.7715
	Erythrocytes	8.76 ± 0.28 a	8.54 ± 0.52 a	8.99 ± 0.32 a	8.87 ± 0.5 a	8.93 ± 0.68 a	0.4648
	Leukocytes	12,714.29 ± 1573.14 b	21,042.86 ± 7104.89 a	14,871.43 ± 4525.38 ab	12,214.29 ± 2664.23 b	15,228.57 ± 4214.15 ab	0.0065
	Lymphocytes	70.14 ± 4.18 a	57.86 ± 16.51 a	58.71 ± 9.34 a	55.14 ± 9.96 a	59.57 ± 8.7 a	0.1055
	Monocytes	4.57 ± 2.44 a	4.57 ± 2.3 a	4.14 ± 1.46 a	3.71 ± 0.95 a	3 ± 0 a	0.3863
	Neutrophils	23.29 ± 0.49 b	37.43 ± 14.64 ab	37 ± 8.76 ab	40.86 ± 9.55 a	37.14 ± 8.28 ab	0.0162
	Platelets	141,342.86 ± 13,792.86 a	145,900 ± 14,369.53 a	125,188.57 ± 28,663.34 a	141,285.71 ± 64,085.49 a	117,728.57 ± 24,191.92 a	0.4908
	RDW	15.71 ± 0.76 ab	16.29 ± 0.76 a	15 ± 0.58 b	16.43 ± 0.53 a	14.71 ± 1.11 b	<0.001
	MCV	67.57 ± 2.99 a	67.29 ± 3.2 a	64.43 ± 1.27 a	66.14 ± 2.85 a	67.57 ± 4.16 a	0.2651
	MPV	6.39 ± 0.72 a	6.94 ± 0.28 a	6.47 ± 0.35 a	6.51 ± 0.57 a	6.66 ± 0.58 a	0.3297
	Body Weight Parameters						
	Spleen	0.99 ± 0.17 a	1.02 ± 0.11 a	0.93 ± 0.16 a	1.02 ± 0.22 a	1.13 ± 0.42 a	0.6567
21 days							
	Hematological Parameters						
	MCHC	21.43 ± 0.79 b	20.86 ± 0.38 b	23.86 ± 0.38 a	23.57 ± 0.79 a	24 ± 0.63 a	<0.001
	MCH	15.86 ± 0.69 a	15 ± 1 ab	15.71 ± 0.49 ab	14.71 ± 0.49 b	15.67 ± 0.52 ab	0.0121
	Hematocrit	64.14 ± 2.85 a	58 ± 18.2 a	54.57 ± 4.69 a	51.43 ± 10.83 a	57 ± 7.24 a	0.2459
	Hemoglobin	13.64 ± 0.52 a	12.21 ± 3.87 a	12.86 ± 1.35 a	11.89 ± 2.27 a	12.32 ± 0.98 a	0.6043
	Erythrocytes	8.67 ± 0.27 a	7.97 ± 2.35 a	8.14 ± 0.71 a	7.81 ± 1.52 a	7.63 ± 0.54 a	0.6714
	Leukocytes	11,414.29 ± 3151.42 a	11,185.71 ± 3943.53 a	12,471.43 ± 2752.4 a	11,471.43 ± 3270.43 a	11,266.67 ± 2073.32 a	0.9408
	Lymphocytes	65.14 ± 3.18 a	67.29 ± 4.75 a	64.86 ± 14.62 a	56.86 ± 5.73 a	61.67 ± 10.54 a	0.2313
	Monocytes	3.57 ± 0.53 a	3.29 ± 0.76 a	3.86 ± 1.95 a	4.86 ± 1.95 a	3.67 ± 0.82 a	0.2762
	Neutrophils	31.29 ± 3.2 a	29.14 ± 4.41 a	31 ± 12.54 a	38.14 ± 4.67 a	34.33 ± 9.93 a	0.247
	Platelets	117,628.57 ± 29,484.67 a	144,242.86 ± 54,778.15 a	133,471.43 ± 31,323.19 a	108,038.57 ± 28,034.55 a	113,566.67 ± 11,992.44 a	0.2917
	RDW	16.71 ± 0.76 a	16.71 ± 0.76 a	15 ± 1.15 b	15.29 ± 1.5 ab	15.5 ± 0.84 ab	0.0082
	MCV	73.71 ± 3.77 a	72.14 ± 3.67 a	64.86 ± 4.45 b	64 ± 1.63 b	66.5 ± 1.87 b	<0.001
	MPV	6.56 ± 0.36 a	6.23 ± 0.36 a	6.64 ± 0.17 a	6.49 ± 0.53 a	6.58 ± 0.58 a	0.4113
	Body Weight Parameters						
	Spleen	1.17 ± 0.21 a	0.96 ± 0.15 a	9.51 ± 22.71 a	1.12 ± 0.16 a	1.18 ± 0.13 a	0.4446

Note: Values are expressed as mean ± standard deviation of the mean. Mean differences were evaluated using ANOVA followed by Tukey’s post hoc test. Different lowercase letters (a, b) within the same row indicate statistically significant differences between groups (*p* < 0.05), whereas values sharing at least one common letter do not differ significantly.

**Table 4:** In silico pathway-, target-, and docking-based biological support for the observed hematological outcomes.

Compound	Reported Molecular Targets *	Docking Binding Affinity (ΔG, kcal/mol) †	Associated Pathways *	Biological Relevance *	Relation to Observed Outcomes	References
*Curcuma longa* (curcumin)	NF-κB, TNF-α, IL-6	−7.5 to −9.3	Inflammatory signaling pathways	Regulation of pro-inflammatory cytokine production and immune stress responses	Consistent with partial preservation of leukocyte and neutrophil counts	[36,37,47]
*Curcuma longa* (curcumin)	Nrf2, ARE-related proteins	−8.0 to −9.1	Antioxidant response pathways	Cellular defense against oxidative stress and protection of hematopoietic progenitors	Coherent with RDW stabilization and attenuation of erythroid dysregulation	[38,47]
*Curcuma longa* (curcumin)	PI3K/Akt, STAT3	−7.8 to −9.0	Cell survival and stress-response signaling	Modulation of cell survival under cytotoxic stress	Compatible with maintenance of erythroid indices during docetaxel exposure	[37,47,48]
*Curcuma longa* (curcumin)	COX-2	−9.0 to −10.2	Inflammatory and immune regulation pathways	Reduction of inflammation-associated tissue stress	Provides biological context for systemic hematological trends	[37,47,48]
*Curcuma longa* (curcumin)	Cytokine signaling nodes	−7.2 to −8.6	Hematopoietic and immune regulatory networks	Adaptive responses under bone marrow suppression	Supports exploratory interpretation of splenic variability	[40,41,47]

* Targets and pathways listed are derived from previously published experimental and in silico studies and curated biological databases. † Docking binding affinity values represent ranges reported in previously published in silico molecular docking studies involving curcumin and the indicated molecular targets.

## Data Availability

The datasets generated and/or analyzed during the current study are not publicly available because they contain detailed organ-toxicity data from individual animals which are subject to institutional ethical restrictions, but are available from the corresponding author upon reasonable request. Requests should be directed to Dr. Isabella Morais Tavares Huber (email: isabellatavares@hotmail.com).

## Abbreviations

The following abbreviations are used in this manuscript:

ANOVA	analysis of variance
ARE	antioxidant response element
CBC	complete blood count
CEUA	Committee for Ethics in the Use of Animals
CONCEA	National Council for the Control of Animal Experimentation
COX-2	cyclooxygenase-2
DTX	docetaxel
GM-CSF	granulocyte–macrophage colony-stimulating factor
Hb	hemoglobin
Hct	hematocrit
HED	human equivalent dose
IACUC	Institutional Animal Care and Use Committee
IKK	IκB kinase
IL	interleukin
IL-6	Interleukin-6
mCRPC	metastatic castration-resistant prostate cancer
MCH	mean corpuscular hemoglobin
MCHC	mean corpuscular hemoglobin concentration
MCV	mean corpuscular volume
MPV	mean platelet volume
NF-κB	nuclear factor kappa B
NK	natural killer
NLR	neutrophil-to-lymphocyte ratio
Nrf2	Nuclear factor erythroid 2-related factor 2
p-Akt	phosphorylated Akt
PI3K	phosphoinositide 3-kinase
PLT	platelets
PTEN	phosphatase and tensin homolog
RBC	red blood cells
RDW	red cell distribution width
ROS	reactive oxygen species
RTKs	receptor tyrosine kinases
SMR	specific metabolic rate
STAT3	Signal transducer and activator of transcription 3
TNF-α	tumor necrosis factor alpha
UNIPAR	Universidade Paranaense
WBC	white blood cells
WHO	World Health Organization
